# Meteorological Register for May, 1841

**Published:** 1841-06-05

**Authors:** J. Conrad


### METEOROLOGICAL REGISTER FOR MAY, 1841.

*Kept at the Pennsylvania Hospital, by J. Conrad.*

| Date. | Thermometer. | | | Barometer. | | Dew Point | Winds. | | Rain. | REMARKS. |
|---|---|---|---|---|---|---|---|---|---|---|
| | Max. | Min. | 9 AM. | 10 AM | 3 PM. | | Direction. | Force | | |
| 1 | 54 | 44 | 51 | 29.59 | 29.62 | 35 | NW. | 3 | | Morning cloudy; aft'n clear. |
| 2 | 63 | 36 | 50 | 29.71 | 29.72 | 30 | SW.NW. | 5 | .080 | Rain in morn; gust at 1½ PM; afternoon clear. |
| 3 | 51 | 32 | 39 | 29.97 | 29.92 | 18 | NW. | 3 | | Clear. |
| 4 | 51 | 35 | 43 | 29.97 | 29.91 | 23 | NW. | 2 | | Clear. |
| 5 | 56 | 36 | 52 | 29.96 | 29.88 | 38 | SE. | 2 | .385 | Clear in morn; rain aftn'n and evening. |
| 6 | 60 | 45 | 53 | 29.89 | 29.94 | 34 | NW. | 3 | | Clear. |
| 7 | 60 | 42 | 54 | 30.16 | 30.11 | 36 | NW.NE. | 1 | | Morn clear; afternoon cloudy; evening rain. |
| 8 | 57 | 45 | 51 | 29.98 | 30.06 | 42 | NE.NW. | 1 | .740 | Rain till 7½ AM; clear at 5 PM. |
| 9 | 68 | 42 | 59 | 30.22 | 30.16 | 40 | NW.SW. | 1 | | Morning clear; aft cloudy. |
| 10 | 66 | 50 | 55 | 29.74 | 29.58 | 56 | SE.SW. | 1 | .510 | Rain till 10 AM; rain from 12 to 2, and 6 to 7 PM. |
| 11 | 67 | 54 | 63 | 29.52 | 29.53 | 48 | SW.NW. | 2 | | Clear. |
| 12 | 60 | 50 | 56 | 29.60 | 29.62 | 48 | W.NW. | 2 | .166 | Showers all day; partly clear. |
| 13 | 65 | 46 | 57 | 29.83 | 29.80 | 41 | NW.NE. | 1 | .115 | Partly clear; rain in even'g. |
| 14 | 62 | 43 | 50 | 29.88 | 29.88 | 36 | NW. | 2 | .005 | Partly clear; sprinkling of rain. |
| 15 | 62 | 43 | 55 | 30.07 | 30.05 | 31 | NE.SE. | 1 | | Clear. |
| 16 | 66 | 43 | 62 | 30.12 | 30.10 | 42 | SW.NW.SE. | 1 | | Clear. |
| 17 | 76 | 48 | 63 | 29.82 | 29.72 | 40 | SW.NW. | 4 | .016 | Morn partly clear; gust at 2 PM; aft'n clear. |
| 18 | 67 | 48 | 58 | 29.93 | 29.87 | 29 | NW. | 2 | | Clear. |
| 19 | 64 | 48 | 58 | 29.96 | 29.96 | 36 | N. | 1 | | Clear. |
| 20 | 69 | 46 | 60 | 30.09 | 30.08 | 40 | NW.SE. | 1 | | Clear. |
| 21 | 78 | 49 | 64 | 30.13 | 30.08 | 55 | SW. | 2 | | Clear. |
| 22 | 87 | 57 | 70 | 30.12 | 30.10 | 62 | SW. | 1 | | Morn clear; cl'dy from 3½ PM. |
| 23 | 81 | 68 | 72 | 30.16 | 30.13 | 64 | SW. | 2 | | Morn partly clear; aft clear. |
| 24 | 85 | 63 | 72 | 30.17 | 30.10 | 62 | SW. | 2 | | Clear. |
| 25 | 83 | 64 | 74 | 30.13 | 30.07 | 64 | SW. | 2 | .555 | Morn fair;thundergust at 6 PM. |
| 26 | 73 | 67 | 72 | 30.07 | 30.05 | 63 | SW. | 1 | .160 | Cloudy; rain from 10 AM. to 1½ PM. |
| 27 | 71 | 64 | 70 | 30.10 | 30.10 | 64 | NE.SE. | 1 | .421 | Rain from 7 AM. |
| 28 | 80 | 64 | 70 | 30.16 | 30.11 | 64 | SE.SW. | 1 | .106 | Clear from 11 AM; shower at 5 PM. |
| 29 | 78 | 65 | 70 | 30.11 | 30.06 | 64 | SW. | 1 | | Morn cloudy; afternoon clear. |
| 30 | 67 | 56 | 60 | 30.11 | 30.12 | 52 | NE.E. | 2 | .010 | Rain at 6 AM; eve'g clear. |
| 31 | 71 | 51 | 61 | 30.12 | 30.05 | 40 | NE.SE. | 1 | | Clear. |
| Mean | 67.67 | 49.80 | 59.48 | 29.98 | 29.95 | 45 | | | 3.269 | |

| | | | |
|---|---|---|---|
| Mean temperature, | 58.74° | Clear days, - - | 19 |
| " pressure, | 29.97 inches. | Cloudy, - - | 9 |
| " dew-point, | 45° | Rain, - - - | 3 |

*Winds*—W. to S. 12 days; S. to E. 4½ days; E. to N. 4½ days; N. to W. 10 days.

